# A Communication Infrastructure for the Health and Social Care Internet of Things: Proof-of-Concept Study

**DOI:** 10.2196/14583

**Published:** 2020-02-25

**Authors:** Vincenzo Della Mea, Mihai Horia Popescu, Dario Gonano, Tomaž Petaros, Ivo Emili, Maria Grazia Fattori

**Affiliations:** 1 Department of Mathematics, Computer Science and Physics University of Udine Udine Italy; 2 Cimtech Srl Reana del Rojale Italy; 3 MIPOT SpA Cormons Italy

**Keywords:** health services for the aged, remote sensing technology, sensors and actuators, embedded systems, Internet of Things, LoRaWAN

## Abstract

**Background:**

Increasing life expectancy and reducing birth rates indicate that the world population is becoming older, with many challenges related to quality of life for old and fragile people, as well as their informal caregivers. In the last few years, novel information and communication technology techniques generally known as the Internet of Things (IoT) have been developed, and they are centered around the provision of computation and communication capabilities to objects. The IoT may provide older people with devices that enable their functional independence in daily life by either extending their own capacity or facilitating the efforts of their caregivers. LoRa is a proprietary wireless transmission protocol optimized for long-range, low-power, low–data-rate applications. LoRaWAN is an open stack built upon LoRa.

**Objective:**

This paper describes an infrastructure designed and experimentally developed to support IoT deployment in a health care setup, and the management of patients with Alzheimer’s disease and dementia has been chosen for a proof-of-concept study. The peculiarity of the proposed approach is that it is based on the LoRaWAN protocol stack, which exploits unlicensed frequencies and allows for the use of very low-power radio devices, making it a rational choice for IoT communication.

**Methods:**

A complete LoRaWAN-based infrastructure was designed, with features partly decided in agreement with caregivers, including outdoor patient tracking to control wandering; fall recognition; and capability of collecting data for further clinical studies. Further features suggested by caregivers were night motion surveillance and indoor tracking for large residential structures. Implementation involved a prototype node with tracking and fall recognition capabilities, a middle layer based on an existing network server, and a Web application for overall management of patients and caregivers. Tests were performed to investigate indoor and outdoor capabilities in a real-world setting and study the applicability of LoRaWAN in health and social care scenarios.

**Results:**

Three experiments were carried out. One aimed to test the technical functionality of the infrastructure, another assessed indoor features, and the last assessed outdoor features. The only critical issue was fall recognition, because a slip was not always easy to recognize.

**Conclusions:**

The project allowed the identification of some advantages and restrictions of the LoRaWAN technology when applied to the health and social care sectors. Free installation allows the development of services that reach ranges comparable to those available with cellular telephony, but without running costs like telephony fees. However, there are technological limitations, which restrict the scenarios in which LoRaWAN is applicable, although there is room for many applications. We believe that setting up low-weight infrastructure and carefully determining whether applications can be concretely implemented within LoRaWAN limits might help in optimizing community care activities while not adding much burden and cost in information technology management.

## Introduction

### The Scenario

Increasing life expectancy and reducing birth rates indicate that the world population is becoming older, with many challenges related to quality of life and well-being for old and fragile people, as well as their informal caregivers, particularly when functional independence decreases and there is a need to provide care on a daily basis. One of the conditions associated with most of the assistance burden is dementia.

In Italy, about 1.2 million people present with some form of dementia, and of these, about half are diagnosed with Alzheimer disease [1]. Patients may experience different levels of cognitive impairment. In the initial stages, they often are free to move around; however, they may not always be able to find their way. In fact, getting lost behavior is present in about 40% of patients with Alzheimer disease [2].

In previous years, Global Positioning System (GPS)–based technologies have been used to develop systems to support patients with Alzheimer disease and dementia in the stages of moderate impairment [3,4] by allowing caregivers to track them. Smartphones can be used for this approach; however, battery capacity and coverage have been reported as issues [3]. Systems have also been developed to work inside buildings [5]. Data collected from tracking systems appear useful to evaluate the evolution of the disease (eg, measure life space [6] and evaluate gait and balance [7]). Furthermore, recent systems allow tracking of people and, in principle, may provide guidance and support to moderately impaired patients [8].

In the last few years, novel information and communication technology techniques generally known as the Internet of Things (IoT) have been developed, and they are centered around the provision of computation and communication capabilities to objects, including those of common usage in daily life. By referring to the above-mentioned scenario, the IoT may provide older people with IoT objects that enable their functional independence in daily living by either extending their own capacity or facilitating the efforts of their caregivers while preserving and increasing, if possible, their functional independence in activities of daily living.

This aim could be achieved through an integrated infrastructure of smart objects with sensors, actuators, and intelligence that populate the homes of older community-dwelling individuals for their own use or caregiver use. These smart objects may include activity/presence sensors, vital sign readers, domotic actuators, and position trackers and may be based on different technologies like traditional sensors, as well as vision-based activity classifiers.

### Internet of Things Requirements

The IoT is a paradigm used to describe “a variety of things or objects, such as radio-frequency identification tags, sensors, actuators, mobile phones, etc, which, through unique addressing schemes, are able to interact with each other and cooperate with their neighbors to reach common goals” [9]. This will be possible only when devices intercommunicate or at least communicate with some application able to receive and eventually send data.

For both data processing and communication, available resources might not always be sufficient, particularly regarding power and bandwidth. In fact, although some IoT objects can be connected to the electric grid as they are indoors and static, some others may be isolated and may require a battery. In this case, battery optimization and durability are strict requirements to guarantee long device backup. Consumption is dependent on processing power, sensors, and communication; thus, optimization needs to address these areas.

As the main aspect of this article is communication, we will focus on the communication infrastructure. In recent years, there has been rapid growth in proposals for low-power wide-area technologies. Although some very low consumption protocols already exist (Bluetooth Low Energy and ZigBee), their low consumption is achieved by substantially restricting the coverage of the transmitter. On the other hand, some IoT applications are deployed in wide areas, namely smart cities, fields, etc, where short range is not a viable option.

There are many technologies and standard proposals that address these needs, and the following three are prominent but different in their approaches:

- Sigfox: It is a network operator that manages a proprietary network based on unlicensed bands (eg, 868 MHz in Europe, 915 MHz in America, 433 MHz in Asia, and 915/923 MHz in Australia). It currently covers about 60 countries. There is no need for deployment and own infrastructure. However, if there is no coverage, it cannot be used. The business model is similar to that of cellular networks. There is a fee for using the network. Additionally, there are limits on message length and the daily number of messages sent. Moreover, the data rate is very limited (100 bits/s); however, this allows for a very long range (>40 km). As it uses free bands, quality of service is not guaranteed [10].
- LoRaWAN: It uses the same bands as Sigfox, but there is no central network operator; thus, there is no use fee [11]. Existing networks could be used or developers could create their own infrastructure. Although limits on data transfer are present, they are less strict than those in Sigfox, and the data rate varies depending on the spread factor (SF; 300 bits/s to 50 kilobits/s). The range is shorter than that of Sigfox (up to 20 km). Similar to Sigfox, quality of service is not guaranteed.
- NBIoT: It is the IoT technology of cellular network operators, exploiting the same licensed bandwidth and infrastructure as phones, with a similar business model [12]. It allows for guaranteed quality of service at a cost. The message length and data rate are substantially higher than those of Sigfox and LoRaWAN. However, the node distance from the base station is less than 10 km.

Although all these technologies are made for the IoT, we selected LoRaWAN because of its ease of implementation, flexibility, and cost, as documented in a previous report [13], where a detailed comparison from many points of view can be found.

### LoRa and LoRaWAN

LoRa is a proprietary wireless transmission protocol that is optimized for long-range, low-power, low–data-rate applications and is developed by Semtech (Camarillo, California) [14]. LoRa uses a proprietary spread spectrum modulation derived from chirp spread spectrum technology [15]. This allows LoRa to increase sensitivity by selecting the amount of spread used according to the radio parameter SF, in the range of 7 to 12. With an increase in sensitivity, the data rate decreases but longer distances can be reached. In addition, forward error correction is implemented, and this further improves robustness against noise. With an allocated bandwidth of 125 kHz, the data rate ranges from 250 to 5470 bits/s, corresponding to a minimum received signal strength indicator (RSSI) of −135.5 to −122.5 and a maximum payload size per packet of 59-230 bytes. This makes it clear that LoRaWAN is not made for transferring large amounts of data.

The frequency range in which LoRa operates has limits that often depend on national regulations, but these are typically aimed at limiting frequency occupation both in terms of transmission time and power. This is because being an unlicensed band, there is no restricted access, unlike in cellular telephony. For example, in Europe, the maximum transmission power is 14 dBi and the maximum duty cycle is between 1% and 10% depending on the specific frequency. National or international regulations specify other limits within unlicensed bands that will not be dealt with in this paper.

In a country where it is mandatory to respect the duty cycle limit, using a low data rate limits the number of packets that can be sent. Power consumption also depends on transmission time, so it is typically advised to select the highest data rate at which transmission succeeds. It should be noted that a LoRa receiver is able to discriminate parallel transmissions at different SFs, thus allowing better exploitation of the band.

LoRaWAN is an open stack built upon LoRa, which defines the communication protocol and system architecture for the network [11]. LoRaWAN takes into account different aspects including the following:

- Security: Packets are encrypted using AES128, with two ways of joining the network (over-the-air activation and activation by personalization), and there is frame counter management to enforce security.
- Optimization of airtime: In LoRaWAN, the network server may recognize when the node is transmitting at a nonoptimal data rate (according to RSSI) and send commands to the node to switch to a faster rate, with a function called adaptive data rate, or to decrease the power used for transmission.
- Application management: The network server associates a node to an application and forwards packets to it, eventually performing some conversion from raw bytes to JSON or another format.

These functions involve a cost to packet length. LoRaWAN adds a header of 13 bytes to be able to deal with the above aspects and sends extra downlink packets to implement the adaptive data rate. For example, an 8-byte payload needs to be added to the 13-byte LoRaWAN header, resulting in a total of 21 bytes.

LoRaWAN defines data rates as specific configurations of SF and bandwidth on a regional basis and depending on the band. The bands are defined in terms of region and central frequency in MHz of the unlicensed spectrum. For example, EU868 refers to the European Region and frequencies ranging from 867.1 MHz to 869.525 MHz. In the European Region, the above-mentioned 21-byte packet will need from 28 ms (at SF7) to 1482 ms (at SF12) of airtime for transmission.

The typical architecture of LoRaWAN implementation is based on the following components:

- Node: It is a physical device, possibly with sensors, which can send and eventually receive packets to and from one or more gateways. In fact, nodes are not tied to a single gateway.
- One or more gateways: It principally receives LoRaWAN packets from nodes and forwards them through the Internet to a network server in a standardized format. It should be noted that nodes are not associated with a specific gateway, and their packets can be received by any gateway in the network.
- Network server: It receives packets from gateways, recognizes duplicate packets, requests nodes to change their data rate, decides to which application packets are directed, may perform some transformation of the payload, and finally sends packets to the selected application.
- One or more applications running on application servers that receive data from the network server.

**Figure 1:**
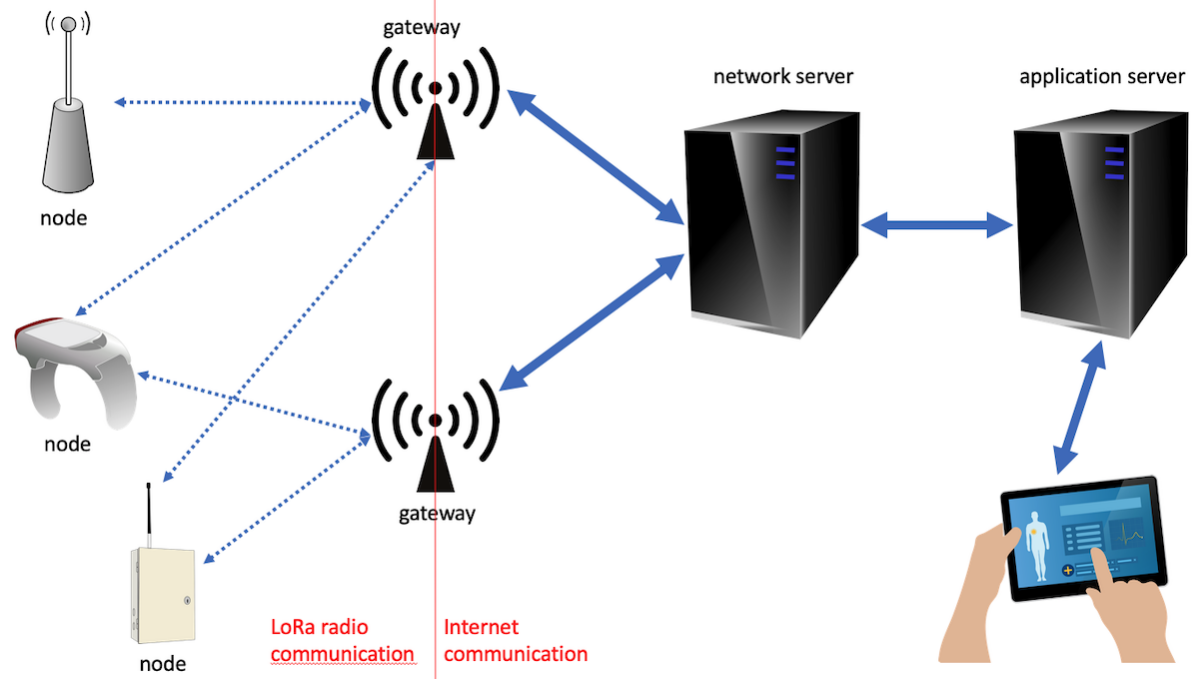
Interactions among LoRaWAN components.

Figure 1 shows the interaction among these components.

As low power consumption is an important requirement, LoRaWAN nodes can be of three types, with functionality constraints that influence the power needed, as follows:

- Class A: Nodes mostly send packets and are able to receive only just after having sent a packet in two receive windows defined by the LoRaWAN standard (ie, 1 s and 2 s after delivery).
- Class B: Nodes may receive data in further slots of time defined and communicated by the network server through the gateway.
- Class C: Nodes are constantly listening for packets.

The most used nodes are Class A, because they represent the typical sensors sending data to an application, with no or limited need for receiving data.

The reported applications of LoRaWAN include agriculture [16], smart cities [17], environmental monitoring [18], and asset tracking and monitoring [19], and in general, it can be used for any form of remote monitoring.

Regarding the health domain, only few previous reports can be found, and most are from conference proceedings. Catherwood et al have described a portable diagnostic reader for urinary tract infection diagnosis that has been connected by means of LoRa [20]. Buyukakkaslar et al have investigated LoRaWAN as a possible electronic health technology but without addressing a specific scenario [21]. Petäjäjärvi et al have framed their performance analysis in a scenario involving a person moving inside a confined and relatively small area both outdoors and indoors [22]. Mdhaffar et al. have performed a similar evaluation but in a larger geographical area [23]. The latter two papers provide useful insights for our experimentation.

### Objectives

This paper describes a LoRaWAN-based infrastructure designed and experimentally developed to support IoT deployment in a health care setup, for which the management of patients with Alzheimer disease and dementia has been chosen as the experimentation field. The designed infrastructure covers all aspects, from physical devices to the application layer, partly exploiting existing technology and developing new modules. With the specific field of dementia management in mind, we developed a wearable device that is able to cover useful functions like localization and fall detection and a Web application for management, patient association, caregiver assignment, etc. The overall system is designed to be easily administered by both specialized and nonspecialized personnel, such as retirement home and hospital employees and patient relatives.

In the below sections, we will provide details on the technologies adopted in this project.

## Methods

### Project Aims

The above-mentioned infrastructure has been adopted in an industrial research project funded by the European Regional Development Program and Regional Operational Program framework through the Regional Government of Friuli-Venezia Giulia. The project entitled “Localization platform for people with cognitive disorders and dementia (PollicIoT)” had the following three main aims: (1) develop a complete system for outdoor patient localization from the hardware to the management platform; (2) ensure that the system can recognize and notify falls; and (3) ensure that the system has the potential for further clinical studies regarding the relationship between motion features and disease stage.

The project partners included an IoT system integrator (Cimtech, Reana del Rojale, Italy; coordinator), a hardware developer (MIPOT SpA, Cormons, Italy), the Medical Informatics & Telemedicine Lab at the University of Udine (responsible for platform design and data analysis), and a social care public company acting as a reference user group (ASP Moro, Codroipo, Italy).

During the project, the following further requirements were suggested by caregivers: (4) provide surveillance for patients subjected to night wandering and (5) provide indoor localization when patients are hosted in large structures.

Although each project aim is not novel by itself and has been implemented in some other system, in this case, the novelty lies in the communication infrastructure used to integrate all approaches.

The design process always involved all technical partners and needed the final users every time. The following sections provide details on the developed components.

### The Node

The device given to patients was designed around a LoRaWAN chip previously developed by one of the project partners (MIPOT SpA). The node includes a GPS and an accelerometer with a barometric altimeter, and it was enriched with flash memory and a Bluetooth transceiver to fulfill the requirements (3) and (5), respectively.

Among the specific requirements for the node, small size and low weight were crucial, because it had to be worn by patients without disturbance. The final size is about 45×87×14 mm and weight is 55 g, and it includes a lithium polymer battery that could theoretically provide up to 1 week of use. Another specific requirement was to have the external surface as simple and anonymous as possible, avoiding buttons if possible. Thus, the node is black and smooth, with no light-emitting diodes and no switches. Its status can be recognized only through the platform, and it starts and remains on when charged. The prototype is charged through universal serial bus.

**Figure 2:**
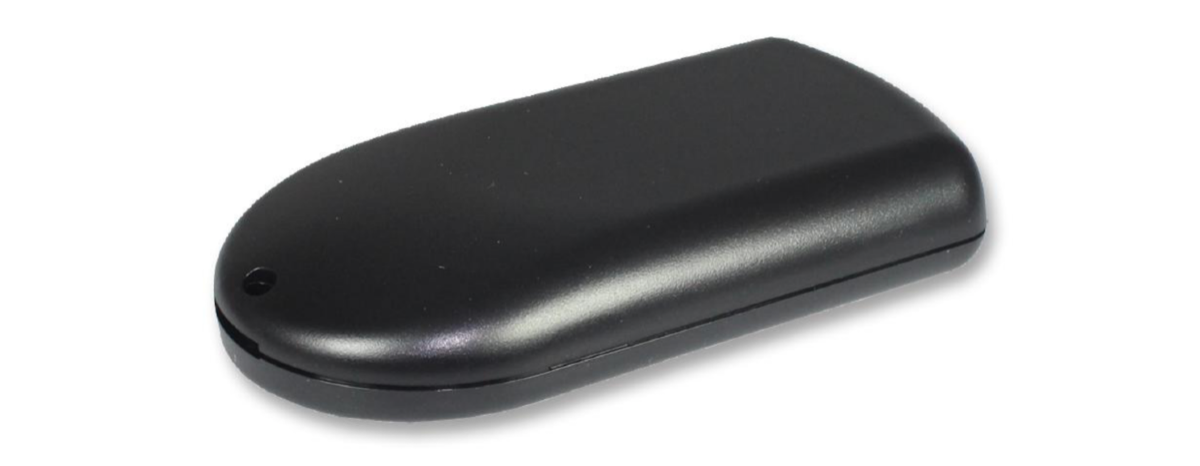
The PollicIoT wearable device.

Figure 2 shows the node prototype. According to the LoRaWAN specifications for moving nodes, the node was set to send packets at a fixed SF of 12, which is the slowest setting, but it provides extended range. This value could be reconsidered depending on the range of the patient. For the experiment, the device was given to patients after agreement with their relatives, and it was worn by means of an armband specifically prepared by the retirement home personnel.

To follow-up on a specific request of the users (requirement 4: night surveillance), towards the end of the project, we implemented a motion detector node, where the LoRaWAN transceiver is installed with an infrared sensor. This node can be placed in the rooms of patients who are known to wander at night, and it seamlessly integrates with the already available infrastructure.

### The Internet of Things Infrastructure

Two gateways were installed for the project. One was placed at the coordinator site for the first functionality test and further roaming tests, and another was placed at the retirement home of the final users.

Regarding the network server, different platforms are already available on the market, and they almost always provide a free tier with some limitations and paid versions with more features or performance. The choice was The Things Network [24], which is a recent yet very active project based on an open source core that, in principle, could be directly used, although it does not have a graphical interface. Initial tests were conducted on the community edition, which is free but has a fair access policy that restricts the time-on-air per device and the number of downlink messages. The final implementation was flawlessly moved to the commercial cloud version.

The platform facilitates the integration of applications by providing a way to decode the binary payload sent by nodes to an application-specific JSON format, which is sent directly to a user-specified URL identifying the application server where the application is running.

### The Application

The user interface of the PollicIoT system involves a Web platform. The core of the system receives JSON messages from the IoT platform, and based on the messages, it localizes the associated patients on a map, sends alarms, etc.

The following features have been implemented:

- Two-level geofencing: Alerts are sent whenever a patient exits one or more safety zones. These zones can be directly drawn on a map inside the platform and can be at the level of the structure hosting the patient (thus valid for every tracked patient) or specific for a single patient (eg, his/her relative’s home and surroundings).
- Multichannel alerts: Alerts about geofencing, falls, battery exhaustion, etc, can be received on the Web platform, as well as through short message service messages and Telegram. The latter functionality has been implemented by means of a Telegram bot.
- Flexible structure management: The system may host one or more organizations, each of which may independently define one or more structures, corresponding to buildings, services, etc. Caregivers belong to a structure, and this allows direction of alerts to the most appropriate caregivers.

**Figure 3:**
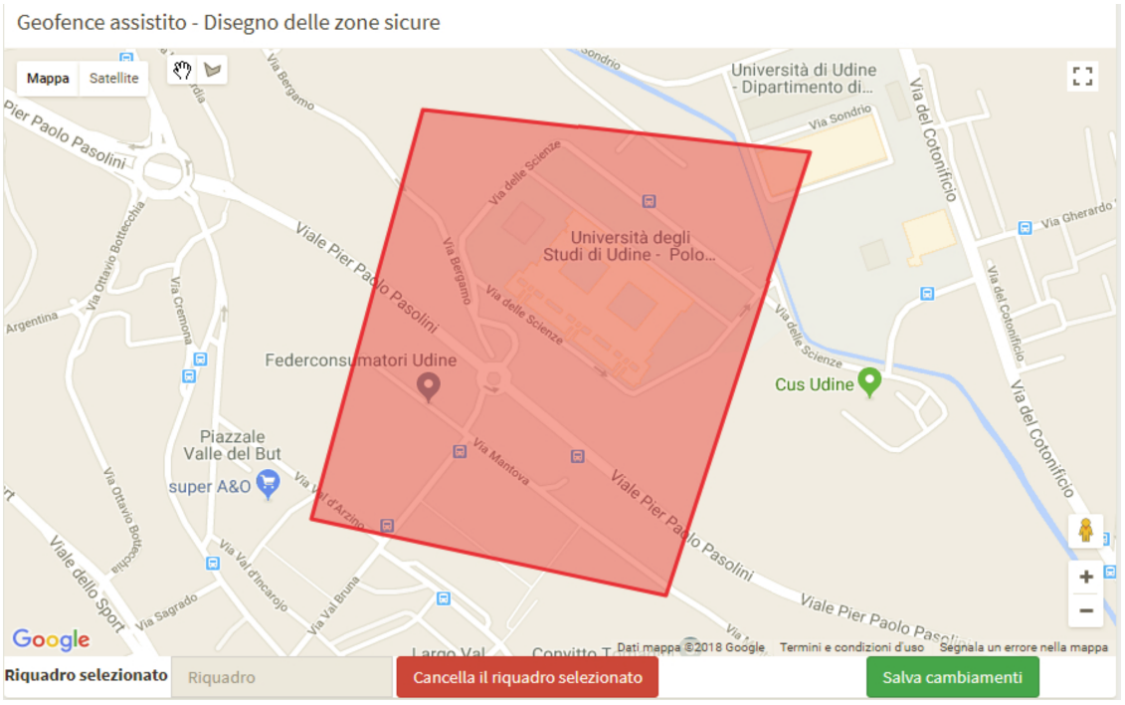
Screenshot of the geofence editor.

**Figure 4:**
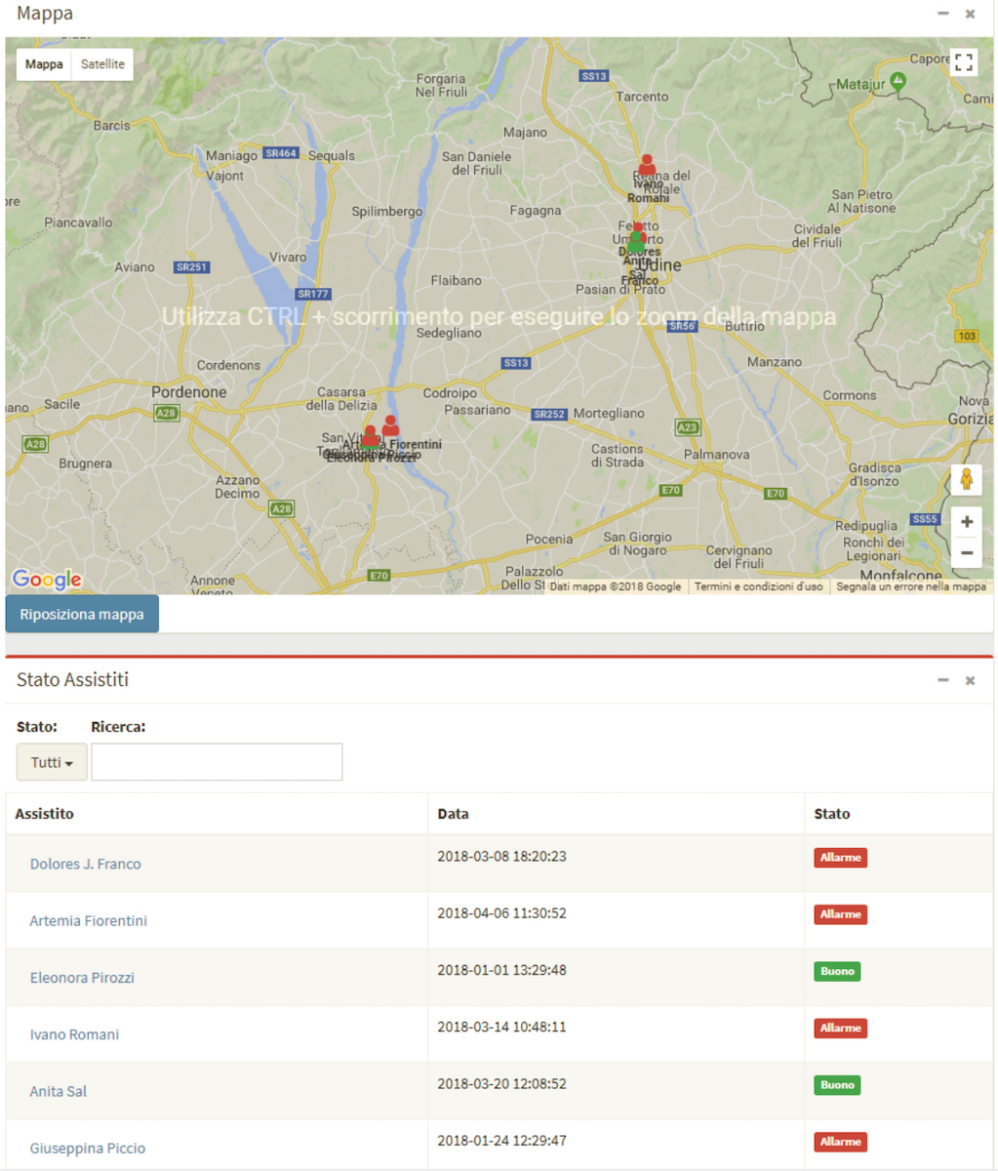
Screenshots of the surveillance map and patient status list.

Figures 3 and 4 show screenshots of two sections of the Web application.

## Results

### Technical Functionality

Three experiments were carried out. The first experiment was related to the evaluation of the technical functionality of the device and the infrastructure. For verification, two authors of this paper used the prototype device for more than a month, freely moving around, including travelling by car, although there is a report on the speed limit under which LoRaWAN is known to function at its best [25]. This allowed demonstration of the robustness of the involved systems and measurement of the maximum distance that could be reached from the gateways (up to 30.5 km in our experiment).

Thereafter, two experiments were aimed at evaluating the following two main application scenarios for patients still living at home: inside a community structure and in the town around the structure.

### Residential Care Scenario

The former scenario involved a small number of patients who carried the wearable device inside a retirement home and nurses who accessed the Web platform for checking positions and alarms, with most movements made within the building. The experiment highlighted that when the device is far from a window, the GPS is not able to provide a position. This was foreseen, and the developed hardware was already designed to have Bluetooth-based localization for the indoors, although the necessary infrastructure was not developed because it was outside of the project.

The fall recognition component was based on the free fall library made available by the company producing the accelerometer chip (STMicroelectronics, Geneva, Switzerland). Although the library can be customized by means of two thresholds (acceleration and altitude before and after the event), it does not appear to adequately capture the kinds of falls elderly people experience, including sliding from chairs, wheelchairs, and beds. In fact, two physiotherapists simulated typical falls while wearing the device, and the fall recognition module was not able to provide reliable alerts. Even the publicly available fall dataset [26] had data recorded during simulated falls among young people; thus, it might be difficult to use the data to develop a reliable elderly fall recognition algorithm. As the collection of true data about elderly falls is extremely difficult, the development of a new fall recognition module has been postponed.

### Home Care Scenario

In the latter scenario, seven social workers carried around the wearable device to map the coverage of the gateway in their work area. This allowed the finding that nodes can communicate in a radius of up to 12 km around the gateway, which was considered sufficient to cover most of the patients in need. The radius obtained does not represent the maximum distance reachable by the nodes, because it is limited by the work area of the social workers, but the finding suggests the feasibility of using LoRaWAN in a typical setting. The map of points has not been included to maintain the privacy of the individuals involved.

**Figure 5:**
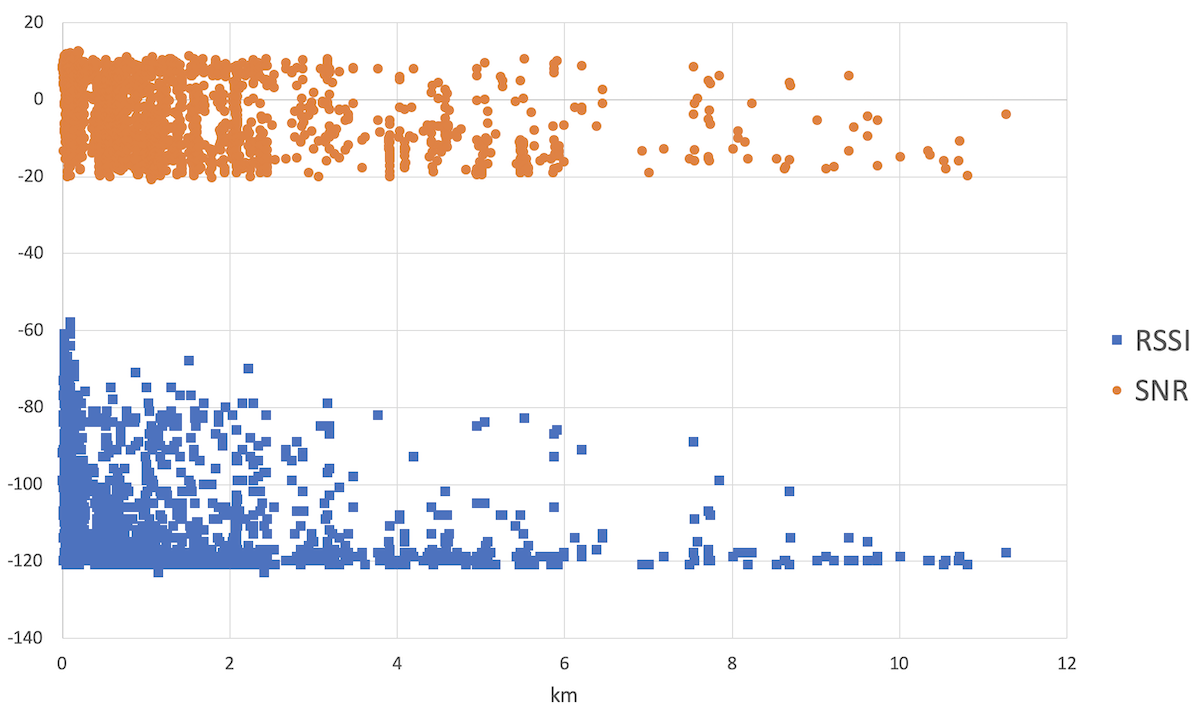
The received signal strength indicator (RSSI) and signal-to-noise ratio (SNR) of the packets received by the gateway.

Figure 5 shows the RSSI and signal-to-noise ratio (SNR), which are measures of signal intensity and quality, plotted according to the distance from the gateway and obtained from 3149 points collected during social worker trips. The plotted points were selected from a total of 3165 points, excluding those that were received by the other gateway in the network.

The variabilities of the RSSI and SNR are high, because they depend on not only distance but also other geographical and environmental factors that cause path loss. The maximum distance recorded in this experiment and toward the main gateway was 11.27 km, although some packets were received by another gateway at 18.63 km.

## Discussion

This project allowed the identification of some advantages and restrictions of the LoRaWAN technology when applied to the health and social care sectors.

Free installation allows the development of services that reach ranges comparable to those available with cellular telephony, but without the need for managing and paying for communication. Although some of these services can be implemented using Wi-Fi inside a retirement home, they cannot be implemented outside, and LoRaWAN appears to be a sensible choice, particularly when dealing with elderly or disabled people who do not necessarily have an Internet connection at home. The following scenarios and applications could be envisaged:

- Social services could identify people in need of remote support, who do not have proper Internet infrastructure at home.
- Social services could identify the nodes needed. For example, a button for signaling events or requests [27]; a device that reminds about pill time; a wearable device that alerts in case of falls; a wearable device that collects data on vital signs like heart rate, steps, etc; and an activity monitor that signals the use of a television, coffee maker, etc.
- The needed nodes could be brought to the homes of patients, and they could be allowed to communicate without any extra infrastructure owned by the host.

Some of the functionalities described above, particularly collection of vital signs, could be implemented through Bluetooth-enabled devices that use LoRaWAN nodes as a bridge to the network, thus extending their original reach. However, to ensure full functionality of the described IoT solution, a survey of the transmission of nodes located indoors should be carried out, because the environment (wall size and composition, radiofrequency noise, etc) has a strong influence on radio transmission. This can be recognized by the findings presented in a report [22] for the indoors and another report [23] for the outdoors involving relatively large areas.

The indoor scenario had partial results, because indoor positioning via Bluetooth was not implemented, and this together with elderly fall recognition will be part of future work. LoRa, in principle, allows positioning by triangulation according to timestamps, with sufficient granularity for positioning precision in the order of meters.

Among the limits of the technology, two are crucial. First, with LoRaWAN, there is no assurance of real-time delivery of packets. In the case of conflicts during transmission, the packet might not be delivered in the first attempt, and it might or might not be resent depending on whether it is sent as “confirmed.” However, as confirmation is expensive in terms of network functioning, confirmed packets should be reduced to the minimum needed. Second, there is duty cycle constraint, which differs depending on country, but ranges between 0.1% and 10% maximum bandwidth occupation. Another limitation is the size of the packets, which is restricted like the bandwidth. All these limitations concur to set the following possible boundaries for LoRaWAN application in the health and social care areas:

- LoRaWAN cannot be used for emergency services, where true real-time communication is needed. This has been recognized in a previous report [20].
- LoRaWAN cannot be used to send large datasets, including images and full biosignal sets.
- LoRaWAN cannot be used when information needs to be sent at high frequencies, which in turn, depends on the distance. Nodes close to the gateway may send data at SF7, with a maximum frequency in the order of some seconds, whereas nodes far from the gateway have to send data at SF12, with a maximum frequency in the order of at least a couple of minutes.

These limits, excluding possibly the first one, can be overcome with smart nodes that embed intelligence to reduce transmission and packet dimension to the minimum needed for a specific application. For example, in our implementation of geofencing, the verification of patient position against the geofence is made at the server level; however, it could be performed inside the node, sending GPS positions only when outside the geofence, which would limit the number of packets sent. Another example is related to biosignals. They could be summarized by some indicator that is sent to the network application, and this could be eventually performed only when abnormal.

However, there are a number of use cases in which these limitations do not occur, including occasional alerts (falls, unforeseen movements, tracking during wandering, pressing communication buttons, etc [events that do not occur frequently]) and daily or generally timed collection of basic vital sign statistics like pressure, heartbeat, and activity, where transmission can be controlled and managed.

It should be noted that limitations are always present in best-effort wireless protocols, including Wi-Fi, because the band resource is shared. However, the potential of LoRaWAN for handling collisions and the capability of receiving signals at different SFs on the same frequency makes it relatively robust regarding issues, particularly with an increase in the number of available gateways. As prices are decreasing, extra gateways can easily be used to extend both the reach and reliability of communication. A fair number of cities and towns, particularly in Europe, are already covered by LoRaWAN public gateways as part of The Things Network [28], and there are other approaches available on other networks that do not disclose coverage maps. This infrastructure enables quick setup of feasibility studies, which can eventually be moved to private networks if needed.

Another crucial topic for health and social applications is security. Aras et al described a number of potential security attacks that were possible on v1.0 compliant LoRaWAN networks [29], and these are partly similar for all wireless technologies (ie, jamming techniques), partly linked to bad implementation practices (ie, no frame counter checks or use of activation by personalization), and partly related to physical tampering with the device. However, Butun et al carried out a similar analysis on version 1.1 of the LoRaWAN protocol and found that it addressed many of the security problems previously reported [30], although some new issues might have been introduced. Health and social care scenarios need to take into account these issues.

We believe that setting up low-weight infrastructure for the above-mentioned scenarios and carefully determining whether applications can be concretely implemented within LoRaWAN limits might help in optimizing community care activities while not adding much burden and cost in information technology management.

## Abbreviations

GPS: Global Positioning System
IoT: Internet of Things
RSSI: received signal strength indicator
SF: spread factor
SNR: signal-to-noise ratio
